# Cochlea cell-specific marker expression upon *in vitro Hes1* knockdown

**DOI:** 10.1590/1414-431X2020e10579

**Published:** 2021-05-17

**Authors:** A.C. Batissoco, K. Lezirovitz, D.B. Zanatta, C.R.M.L. Hemza, L.R. Vasques, B.E. Strauss, R.C. Mingroni-Netto, L.A. Haddad, R.F. Bento, J. Oiticica

**Affiliations:** 1 Otorrinolaringologia/LIM32, Hospital das Clínicas, Faculdade de Medicina, Universidade de São Paulo, São Paulo, SP, Brasil; 2 Departamento de Otorrinolaringologia, Faculdade de Medicina, Universidade de São Paulo, São Paulo, SP, Brasil; 3 Laboratório de Vetores Virais, Centro de Investigação Translacional em Oncologia/LIM24, Instituto do Câncer do Estado de São Paulo, Faculdade de Medicina, Universidade de São Paulo, São Paulo, Brasil; 4 Centro de Estudos sobre o Genoma Humano e Células-Tronco, Departamento de Genética e Biologia Evolutiva, Instituto de Biociências, Universidade de São Paulo, São Paulo, SP, Brasil

**Keywords:** shRNA, Hearing loss, Inner hair cells, Organ of Corti, Hes1 knockdown, Lentivirus

## Abstract

NOTCH pathway proteins, including the transcriptional factor HES1, play crucial roles in the development of the inner ear by means of the lateral inhibition mechanism, in which supporting cells have their phenotype preserved while they are prevented from becoming hair cells. Genetic manipulation of this pathway has been demonstrated to increase hair cell number. The present study aimed to investigate gene expression effects in hair cells and supporting cells after *Hes1*-shRNA lentivirus transduction in organotypic cultures of the organ of Corti from postnatal-day-3 mice. Forty-eight hours after *in vitro* knockdown, *Hes1* gene expression was reduced at both mRNA and protein levels. *Myo7a* (hair cell marker) and *Sox2* (progenitor cell marker) mRNA levels also significantly increased. The modulation of gene expression in the organ of Corti upon *Hes1* knockdown is consistent with cell phenotypes related to lateral inhibition mechanism interference in the inner ear. The lentivirus-based expression of *Hes1*-shRNA is a valuable strategy for genetic interference in the organ of Corti and for future evaluation of its efficacy in protocols aiming at the regeneration of hair cells *in vivo*.

## Introduction

Hearing loss (HL) is the most prevalent sensorineural disorder in humans, and one of the main public health concerns worldwide (1). In the cochlea, hair cells (HC) and supporting cells (SC) are the major cell types of the organ of Corti (OC), an extremely sensitive sensory epithelium with sound mechanotransduction properties. The mammalian cochlea harbors few progenitor cells with limited postnatal ability to give rise to both HCs and SCs, as well as to other cell types in this organ (2,3). As sensorineural HL usually relates to permanent HC damage, reactivation of the cell cycle of quiescent progenitor cells and their differentiation into HCs are seen as potential therapeutic alternatives aiming at the recovery of auditory function and improvement of the quality of life of HL patients (4).

NOTCH signaling pathway proteins have crucial roles in maintaining the SC phenotype and preventing their trans-differentiation into HC by means of the lateral inhibitory mechanism (LIM) (5–7). In the inner ear, this mechanism involves NOTCH pathway ligands, expressed by HC, activating the signaling in the neighboring SC, which then express transcriptional factors, including HES1 (hairy and enhancer of split-1), a repressor of SC differentiation into HC (8).

*Hes1* down-regulation by small interfering RNA (siRNA) encapsulated within polylactide-co-glycolide acid (PLGA) nanoparticles increased HC number in organotypic cultures of cochleae and maculae of postnatal-day-3 (P3) mice pretreated with toxins to eliminate HC. In addition, evidence for the transdifferentiation of SC into HC was presented upon Hes1 down-regulation in cultures using cochleae and maculae of Cdkn1b/GFP (green fluorescent protein)-expressing mouse pups. Cdkn1 (or p27kip1) protein is expressed only in SC of the embryonic and postnatal inner ear sensory epithelia, and in this model, GFP expression is observed in all types of SCs but not in HC. In this experiment, the presence of nascent HC coexpressing with the HC marker myosin VIIa and the SC marker cdkn1/GFP was observed (9). Here, we present conditions to knockdown *Hes1* expression in OC cultures using a lentiviral vector and short hairpin RNA (shRNA). Among five shRNA sequences initially screened, we selected two that reduced *Hes1* expression at both mRNA and protein levels. The expression of one sequence, in particular, led to an increase in the HC marker Myo7A mRNA and protein. We opted for using a lentiviral vector in the *in vitro* assays presented here, as it may provide longer-lasting *in vivo* effects for future perspectives.

## Material and Methods

### Animals

The experimental protocol was previously approved by the Internal Review Board on Ethics in Animal Research from the Medical School and the Institute of Biosciences of the University of São Paulo (Process Number: 0466/08). All experiments were conducted in accordance with the guidelines for the care and use of laboratory animals established by the American National Research Council. In this study, we used male P3 BALB/c mice (*Mus musculus*) obtained from Centro de Bioterismo (University of São Paulo School of Medicine, Brazil).

### shRNA plasmid clones

Recombinant DNA plasmid pLKO.1-puro-CMV-tGFP (Mouse MISSION shRNA Plasmid DNA) containing inserts for the expression of shRNA targeting five different regions of mouse *Hes1* mRNA, named I (CloneID: XM_192801.2-286s1c1); II (CloneID:XM_192801.2-365s1c1); III (CloneID: XM_192801.2-387s1c1); IV (CloneID:XM_192801.2-431s1c1); and V (CloneID: XM_192801.2-678s1c1), in addition to a control shRNA plasmid (SHC003), were obtained from Sigma-Aldrich (USA). Each of the five DNA plasmid clones was used to transform bacteria that were further expanded before maxi-purification of plasmid DNA (QIAGEN, USA).

A patent application has been made for the used methodology and the shRNA plasmid clones described in this study (INPI - Instituto Nacional da Propriedade Industrial, Brasil, Registration number: BR1020140199292 A2, Registered on: 08 August 2014).

### Initial assessment of shRNA-based interference efficiency

The efficiency of the target gene expression knockdown was evaluated in NIH3T3 cells (immortalized embryonic mouse fibroblast, kindly provided by M.C. Sogayar, Biochemistry Department of the Chemistry Institute, University of São Paulo). For transient transfection, NIH3T3 cells were cultured for 24 h and then transfected with Lipofectamine 2000 (Invitrogen, USA) and 2.5 μg of plasmid DNA according to the manufacturer's instructions. The cells were transferred to a 10-cm dish with DMEM containing 1 μg/mL puromycin (both from Invitrogen) and, after two weeks of selective culturing, cells were harvested and total RNA extracted. The plasmid vector carrying the shRNA transgene also has the genes for puromycin resistance and for expression of Turbo Green Fluorescent Protein (tGFP or TurboGFP). For cell viability assessment, 2×10^3^ NIH3T3 cells per well of a 96-well dish were transfected with no DNA or with plasmid DNAs for scrambled control, Hes1.I and Hes1.II clones, employing six individual wells for each group. Cell viability was assessed 48 h later with Cell Proliferation kit II (XTT; Merck, Germany) with replicates A and B. Results were acquired by absorbance at 550 nm having 650 nm as the reference wavelength in a Synergy H1 microplate spectrophotometry reader (BioTek, USA). The mean values for control or experimental groups were submitted to pairwise comparisons using the *t*-test and significance was accepted if P<0.05.

### Lentiviral subcloning, packaging, and particle production

The lentiviral pLKO.1-puro-CMV-tGFP vector (Sigma-Aldrich) was used to subclone the insert sequences I and II yielding, respectively, the recombinant clones Hes1.I (Species: XM_192801.2-286s1c1/Alternative species: NM_008235.2.XM_001000689.1/TRC1trcn0000028927) and Hes1.II (Species: XM_192801.2-365s1c1/Alternative species: NM_008235.2.XM_001000689.1/TRC1 trcn0000028855). The control plasmid (SHC003) was obtained from Sigma-Aldrich. Each clone harbors the TurboGFP (tGFP) reporter gene cDNA. To produce virus particles, each lentiviral plasmid vector was co-transfected with the pCMV-VSV-G and psPAX2 packaging vectors (Addgene, USA) by the calcium phosphate precipitation method in 293T cells (ATCC, USA). Viral supernatants were enriched by ultracentrifugation at 65,000 *g*, for 90 min, at 4°C (SW32 Ti rotor, Beckman Coulter, USA,) and stored at -80°C. Vector titers were determined in mouse fibroblast NIH-3T3 cells (ATCC) by flow cytometry analysis of GFP expression and quantified as number of transducing units (TU) per milliliter.

### Organotypic cochlear sensory epithelium culture and transduction procedure

After dissection (10), the OC epithelia were transferred to a 48-well plate previously coated with 0.01% poly-L-ornithine and 50 μg/mL laminin (Sigma-Aldrich). After culture for 24 h in specific medium (10), viral transduction was performed with 1.6×10^5^ TU per organ in a well of a 96-well plate, for 6 h. Medium was changed and the OC was cultured for an additional 48 h (OC explants). The tissue was used for RT-qPCR, slot blotting, or flow cytometry analyses as presented below.

### RNA extraction and quantitative real-time PCR analyses

Total RNA was extracted from transfected NIH3T3 cells or transduced mouse OC explants using the QIAGEN RNeasy mini Kit (Germany) and the cDNA synthesis was performed with SuperScript^®^ III Kit (Life Technologies, USA). Primers were designed specifically for mouse *Hes1*, *Myo7a*, *Sox2*, and *Cdkn1b* genes (Table 1). PCR reactions were carried out in a SYBR green master mix (Life Technologies) with 100 nM of each primer and 1 uL of cDNA, according to the manufacturer's protocol.

**Table 1:** Oligonucleotide sequences for RT-qPCR primers.

Gene	Ref Seq	Forward primer (5′-3′)Reverse primer (5′-3′)	Amplicon
*Hes1*	NM_008235.2	TCCAAGCTAGAGAAGGCAGAC GTCACCTCGTTCATGCACTC	149 bp
*Myo7a*	NM_001256081.1	CAGGCCAGGAGTTTGATGTG GGTGCATTGGCTTGATGTG	135 bp
*Sox2*	NM_011443.3	CAGGAGTTGTCAAGGCAGAGAAG CTTAAGCCTCGGGCTCCAAAC	132 bp
*Cdkn1b* (p27^kip1^)	NM_009875.4	GGTGGACCAAATGCCTGACTC TCTGTTCTGTTGGCCCTTTTG	123 bp
*Tbp*	NM_013684	CCACACCAGCTTCTGAGAGC GACTGCAGCAAATCGCTTGGG	145 bp
*B2m*	NM_009735.3	TCGCGGTCGCTTCAGTCGTC TTCTCCGGTGGGTGGCGTGA	132 bp

Each experiment was performed in triplicate, for which at least one 10-cm plate of NIH3T3 cells or twelve OC (six animals) were used for each of the five oligonucleotide sequences, besides the control vector. We performed three independent experiments to evaluate mRNA levels by RT-qPCR. Control plasmid was used as the reference sample and *Tbp* or *B2m* as the reference gene (Table 1). For each comparison, all triplicate samples for both groups were assayed in the same run. Samples with no cDNA were negative controls for all experiments. RT-qPCR efficiency varied from 1.9 to 2.1. The threshold cycle (Ct) was normalized to the housekeeping *Tbp* or *B2m* genes and the 2^-ΔΔCT^ method was employed to calculate changes in gene expression. All data are reported as means±SE and compared using the one-tailed unpaired *t*-test with significance of 95%.

### Antibodies for immunofluorescence, slot blotting, and flow cytometry analyses

Rabbit polyclonal anti-Hes1 antibody was obtained from Abcam (USA), rabbit polyclonal anti-myosinVIIa antibody from Affinity BioReagents (USA), rabbit polyclonal anti-Connexin 26 antibody from Thermo Fisher Scientific (USA), and mouse monoclonal anti-alpha-tubulin antibody clone DM1A from Sigma-Aldrich. Secondary antibodies were directed to either mouse or rabbit IgG heavy chains, and conjugated to Alexa Fluor 488 (Jackson Immuno Research, USA) or to Alexa Fluor 546 (Invitrogen). Mouse monoclonal antibody anti-GFP was conjugated to Alexa 488 (USA).

### Indirect immunofluorescence

Mouse OC explants were fixed in 4% paraformaldehyde (Electron Microscopy Sciences, USA), for 30 min, at 4°C, and incubated with 30% sucrose, for 16 h, at 4°C. OCs were included in Jung Tissue Freezing Medium (Leica Biosystems, USA) before freezing and cutting histological sections (10 μm) on a cryostat (CM1850, Leica, Germany). The slides containing the histological sections of mouse OCs were permeabilized in 0.3% triton X-100 for 20 min at room temperature, blocked in 10% goat serum (Santa Cruz Biotechnologies, USA), and incubated for 16 h at 4°C with 100-fold diluted anti-GFP antibody conjugated with Alexa 488 in PBS, 3% BSA (Invitrogen). The slides were mounted in ProLong Gold Antifade reagent containing 4',6-diamidine-2-phenyl indol (DAPI, Invitrogen) for nuclear identification. Images were acquired using the LSM510 confocal microscope (Carl Zeiss, Germany).

### Slot blotting

Due to the limited amount of protein obtained from the OC, slot blotting was adopted to determine the protein levels of Hes1 48 h after the lentiviral transduction procedure. For each analyzed condition (Hes1.I shRNA, Hes1.II shRNA, or control vector) at least twelve OC explants were transferred to a microtube containing 100 µL of RIPA buffer containing 1× protease inhibitor (Complete, EDTA-free, Sigma-Aldrich) and the tissue was homogenized using a Douncer homogenizer (Corning, USA) (40 slow strokes). The protein quantity was estimated using a Bradford reagent (Bio-Rad, USA) at 595-nm absorbance.

Slot blotting was performed in 45-µm nitrocellulose filter in the equipment from Bio-Rad following the manufacturer's protocol. Blotting efficiency was observed after 1.5% Ponceau-S staining. Proteins were blocked for one hour in 1% casein (Sigma-Aldrich) followed by 10 min in 3% hydrogen peroxide. Blots were incubated with primary antibody (one thousand-fold dilution of anti-Hes1 and ten thousand-fold dilution of anti-tubulin antibodies used as reference) for one hour, followed by secondary antibody incubation for one hour at room temperature. The filter was incubated in ECL™ Plus (GE Healthcare, USA) and exposed to Amersham Hyperfilm™ ECL film (GE Healthcare). Densitometric analyses were performed using ImageJ 1.38e software (http://rsb.info.nih.gov/ij/) to measure the intensity of the bands.

### Flow cytometry

Flow cytometry analyses were conducted to evaluate the relative number of OC cells expressing Hes1, Myo7a, and Cx26 proteins. A typical experiment using eighteen animals had a pool of twelve OC explants for each analyzed condition (Hes1.I shRNA, Hes 1.II shRNA, and control vector). Forty-eight h after the lentiviral transduction procedure, the mouse OC explants were transferred to a microtube containing 100 μL of DPBS (Dulbecco's phosphate-buffered saline) and 100 μL of 0.25% trypsin/EDTA (both from Invitrogen), and incubated for 15 min at 37°C. Then, 50 μL of trypsin inhibitor and 50 μL of DNAse (both from Invitrogen) were added and the samples were incubated for 15 min at 37°C. The tissues were mechanically dissociated by passage through 200-μL pipette tips (Eppendorf, Germany) and filtered through a 100-μm cell strainer (BD Falcon^TM^, USA) to remove cell debris. Twenty μL of the supernatant were used for cell morphology observation and counting using an Axiovert 40C microscope (Zeiss, Germany).

The cells were fixed in 4% paraformaldehyde (Electron Microscopy Sciences, USA) in PBS for 15 min at 4°C and permeabilized with 0.2% triton X-100 (Sigma-Aldrich) for 10 min at 4°C. Cells were washed once with PBS, blocked with 2% BSA (bovine serum albumin, Invitrogen) for 30 min at 4°C, and incubated for 16 h at 4°C in the presence of the primary antibody in a 50-fold dilution in PBS, 2% BSA. Cells were washed once with PBS and incubated for one hour at room temperature in 500-fold dilution of Alexa Fluor 488-conjugated anti-rabbit secondary antibodies (Jackson Immuno Research) in PBS. Cells were suspended in 300 μL of PBS and analyzed using the 488 LASER channel of FACS Aria II Flow Cytometer and Diva software (BD Biosciences, USA). As statistical analysis could not be performed, changes of at least 0.2-fold as cutoff were considered.

## Results

**Figure 1:**
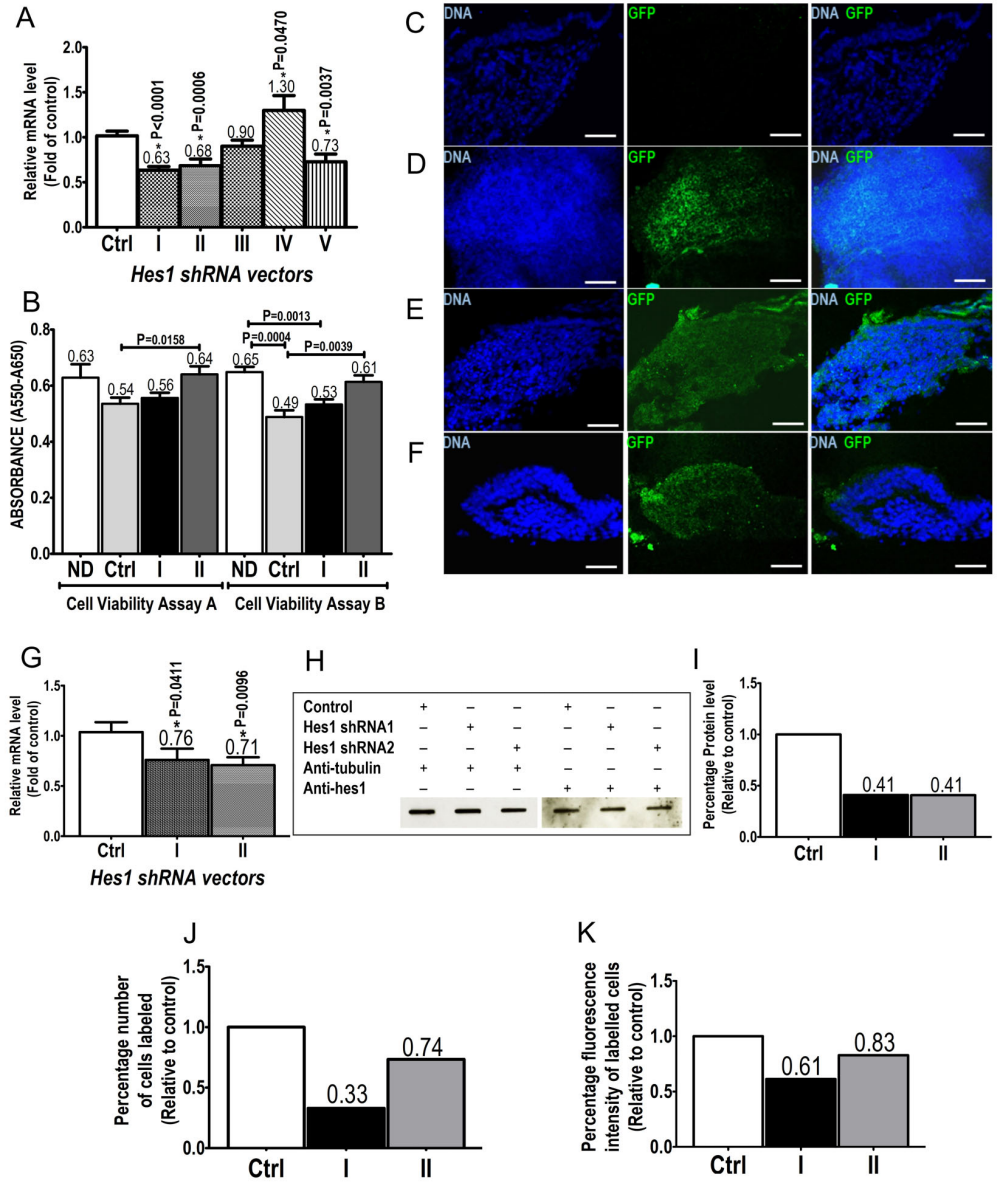
Efficient transfection of plasmid vector and transduction of lentiviral vector with downregulation of Hes1 mRNA and protein levels by shRNA. **A**, RT-qPCR data showing the average fold change of *Hes1* mRNA level in NIH3T3 cells after *Hes1* silencing by each of the five (I-V) *Hes1* shRNA expression vectors or control vector (Ctrl). **B**, Cell viability assay of transfected NIH3T3 with no DNA or plasmid DNAs from scrambled (control), Hes1.I, or Hes1.II clones (n=6/group). The formazan dye produced by metabolically active cells was measured by absorbance at 550 nm normalized to absorbance at 650 nm. **C-F**, Immunofluorescence analysis of organ of Corti (OC) sections stained with anti-GFP (green) antibody: **C**, negative control (without vector transduction); **D**, transduction with control vector; **E** and **F**, transduction with the Hes1.I and Hes1.II shRNA expression vectors, respectively. The nuclei were counter-stained with DAPI (blue). Analysis was performed under a confocal microscope (LSM510, Carl Zeiss). Scale bar: 50 μm. **G**, RT-qPCR results are reported for shRNA assays of organotypically cultured OC with two *Hes1* shRNA expression vectors (I and II) and the control vector (Ctrl). **H**, Slot blot of 64-fold diluted transduced OC lysate, each representing 0.625 µg of total OC protein. Anti-tubulin (reference protein) and anti-Hes1 (interest protein) antibodies were used. **I**, Percentage of densitometric signal intensity of data from panel **G** relative to the control (100%). **J** and **K**, Flow cytometry analysis of OC cells submitted to *Hes1* shRNA knockdown. The percentage number of cells (**J**) and the fluorescence intensity (**K**) according to anti-Hes1 antibody labeling are presented for both control vector and *Hes1* I and II vectors. **G** through **J**, represent data from pools of at least 12 OC explants each. In **A**, **B**, and **G**, data are reported as means±SE. *P<0.05 for each shRNA condition compared to control (*t*-test).

We initially employed NIH3T3 cells and five different shRNA sequences designed to knockdown mouse *Hes1* gene expression. RT-qPCR disclosed *Hes1* mRNA levels significantly lower in cells transfected with shRNA vectors I, II, or V than the control, displaying 37, 32, and 27% reduction, respectively (Figure 1A). Conversely, shRNA vector IV increased *Hes1* mRNA level (*t*-test, n=3, Hes1.IV *vs* control; P=0.047).

The viability of NIH3T3 cells was assessed in replicates after transfection with shRNA vectors Hes1.I or Hes1.II. In both experiments, cells transfected with clone Hes1.II were as viable as those transfected with no plasmid DNA or clone Hes1.I, and more viable than cells transfected with the scrambled shRNA clone. In one experiment, cells transfected with the scrambled shRNA clone or with clone Hes1.I were less viable than those transfected with vehicle only (no DNA), and Hes1.I values corresponded to 82% of those for this negative control (Figure 1B).

As in NIH3T3 cell vectors I and II yielded similar results for *Hes1* knockdown (*t*-test, n=3, Hes1.I *vs* Hes1.II, P=0.2650), each plasmid insert I or II was subcloned into the lentiviral vector pLKO.1-puro-CMV-tGFP, which delivered and expressed the *Hes1* shRNA in cultured organotypic OC cultures for 48 h. Successful OC lentivirus transduction was confirmed by a significant number of cells positive for the reporter Turbo Green Fluorescent Protein (tGFP) (Figure 1C-F), similar to previous reports (11 [Bibr B12]). Both tested constructs (I and II) significantly reduced *Hes1* transcript levels in the OC, compared to the control vector (Figure 1G; *t*-test, n=3, Hes1.I *vs* control; and Hes1.II *vs* control; respectively P=0.041 and P=0.0096). Knockdown rates were similar to those previously observed in NIH3T3 cells (24% for shRNA I and 29% for shRNA II; Figure 1B). Slot blotting disclosed an average 59% reduction of the Hes1 protein by either shRNA clone I or II (Figure 1H and I). FACS assay outputs as labelled cell number or fluorescence intensity disclosed Hes1 protein reductions of 67 and 26.5% of the total number of DAPI-labelled cells, and 39 and 17% of DAPI-normalized fluorescence intensity, respectively for clones I and II compared to the control (Figure 1J and K and Supplementary Figure S1).

**Figure 2:**
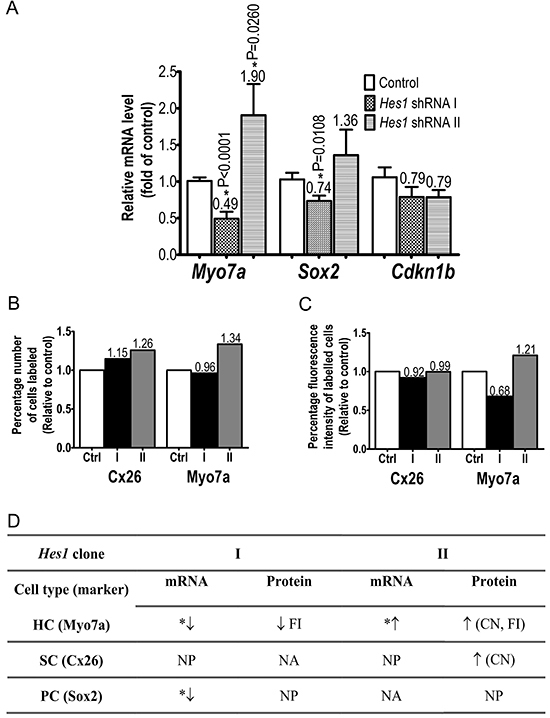
Downregulation of Hes1 mRNA and protein affects organ of Corti (OC) supporting cells (SC), hair cells (HC), and progenitor cells gene marker expression. **A**, *Myo7a*, *Sox2*, and *Cdkn1b* RT-qPCR results after knockdown of OC *Hes1* by shRNA I or II. The data are reported as means±SE. *P<0.05 for each shRNA condition compared to control (*t*-test). **B** and **C**, Flow cytometry analysis of OC cells (pools of at least 12 OC explants) submitted to Hes1 shRNA knockdown. The percentage number of cells (**B**) and fluorescence intensity (**C**) labeled by anti-Myo7a (HC marker) and anti-Cx26 (SC marker) antibodies are presented for control vector and both Hes1 groups. **D**, Table summarizing the effects of *Hes1* knockdown on SC, HC, and progenitor cell (PC) marker expression in the OC. Changes represented in the table as increase (↑) or decrease (↓) are for statistically significant results (*) and, when statistical analysis could not be performed, arrows indicate changes of at least 0.2-fold. CN: FACS fluorescence cell number; FI: fluorescence intensity; NA: not altered; NP: not performed.

In the OC, the HC biomarker *Myo7a* mRNA level had a significant 0.5-fold decrease or 1.9-fold increase after transduction with clones I or II, respectively (Figure 2A and D and Supplementary Figure S2). FACS analyses of the number of cells labelled by the anti-Myo7a antibody corresponded to 0.96 or 1.34 of the control, respectively for clones I or II (Figure 2B and D). Myo7A FACS fluorescence signal intensity upon transduction with clone I or II corresponded to 0.68 or 1.2 relative to the control, respectively (Figure 2C and D).

The progenitor cell marker *Sox2* mRNA quantity significantly decreased upon transduction of OC with clone Hes1.I (1.4-fold) but not with Hes1.II (0.7-fold, Figure 2A and D). No effect was observed for the SC marker *Cdkn1b* at mRNA level. The number of OC cells labelled by FACS for the SC marker Cx26 was above the control in the Hes1-II group (Figure 2B-D, Supplementary Figure S2).

## Discussion

Loss of the inner ear sensory HC, responsible for transduction of sound into electrical synaptic input, is the leading cause of sensorineural HL in humans. Thus, efforts aiming at inner ear regeneration and hearing restoration usually focus on HCs by means of a wide variety of strategies. They consist of overexpression or knockdown of various cell cycle genes, induction of inner ear stem cell proliferation and differentiation in the cochlea, and direct conversion of SCs to HCs by multiple transcription factors. Among them, it is believed that those targeting *in situ* HC renewed proliferation and regeneration should lead to higher odds of auditory function reestablishment, given that the SC and the residual HC reside in their natural sites in the cochlea (2).

Multiple genes and signaling pathways regulate the inner ear HC development. The notch pathway, including the *Hes1* gene, is found to be crucial for normal HC proliferation and differentiation. In the OC, this pathway is activated by HC-delivered notch ligands that bind to their receptor on neighboring undifferentiated SC. HES/HEY basic-loop-helix downstream transcription factors have the role of inhibiting the expression of *Atoh1* and other pro-HC genes. Thus, downregulation of notch signaling pathways is required for HC regeneration. In mammals, more pronounced expression of *Atoh1* is observed upon the emergence of the HCs during embryonic sensory epithelium development and is downregulated after HCs have differentiated. *Atoh1* expression continues low throughout adulthood, both in outer and inner HC. On the other hand, *Hes1* expression becomes elevated at late embryonic and early postnatal stages and is maintained at a relatively high level throughout adulthood in SC, which may be one of the mechanisms that maintain the mosaic pattern arrangement of HC and SC in the OC auditory sensory epithelium (58,). Accordingly, in the absence of *Hes1* function during development, overproduction of HC derived from SC is observed. Moreover, pharmaceutical inhibitors that inactivate notch and consequently reduce Hes1 function at later developmental stages elicit SC to convert into HC, suggesting that notch signaling hold SC fate after differentiation (13–15).

In this study, we individually expressed five DNA sequences as shRNA to target *Hes1* mRNA. Initial assessment of mRNA stability was performed in NIH3T3 cells, disclosing three sequences with *Hes1* silencing capacity (Figure 1A). NIH3T3 cell viability was similar after transfection with clones Hes1.I or Hes1.II. The former was less viable than the vehicle-only control (Figure 1B). Curiously, the scrambled shRNA clone affected cell viability compared to the vehicle control or clone Hes1.II. Since in replicate experiments the negative (scrambled) control reached the lowest values (nearly 25% lower than vehicle control) for cell viability independently on cell numbers, we assumed that the assays presented here for clones Hes1.I and Hes1.II did not reflect cell damage but actual effects on marker expression.

For the OC assays, we selected the two sequences (clones I and II) with the highest reduction rates over *Hes1* mRNA levels. Data were indeed reproducible in the OC, with silencing effects on *Hes1* mRNA similar to those in NIH3T3 cells, in addition to a knockdown effect on Hes1 protein levels. This first set of results confirmed the ability of clones I and II to silence *Hes1* mRNA in the OC by the shRNA-expressing lentiviral vector (Figure 1C-I).

The lentiviral expression of clones I and II led to significantly different OC cell phenotypes. The former decreased *Sox2* mRNA quantity as well as *Myo7a* mRNA and protein levels (Figure 2). Sox2 plays critical roles in cellular reprogramming and stem cell pluripotency. In the development of cochlear cells, the expansion of the progenitors that become HC has been ascribed to *Sox2* expression (16). *Cdkn1b* expression relates to cell mitotic quiescence state, being characteristic of the mature OC SCs (17). It appears that *Hes1* knockdown in the OC for 48 h was not sufficient to impact SC quiescence state since *Cdkn1b* mRNA levels did not change. *Cdkn1b* is downregulated during HC differentiation, but persists at high levels in differentiated SC of the mature OC (17,18).

The expression of *Hes1*-shRNA clone II in the OC led to a significant increase in *Myo7a* mRNA levels and in the ratio of Myo7a protein compared to the control (Figure 2). Myo7a is a specific marker for both inner and outer HC. Although these data indicated that shRNA II was able to affect the expression of the HC marker, it is as yet unclear if the increase in *Myo7A* expression occurred in HC or in cells undergoing transdifferentiation from SC to HC, as described before (16,19). In addition, we may not discard the possibility that the increase in *Myo7a* mRNA and Myo7a protein upon OC transduction with clone Hes1.II could be in part due to cell damage upon expression of the negative (scrambled) control shRNA (Figure 1B). Following the data reported by Du et al. (9) on the effect of *Hes1* RNA interference on increased HC number in murine OC organotypic cultures, a more recent study on adult guinea pigs submitted to noise-induced cochleae injury disclosed functional hearing recovery upon delivery of encapsulated *Hes1*-siRNA (20).

Our study disclosed one out of five *Hes1*-shRNA sequences silencing *Hes1* in the OC from P3 mice affected *Myo7A* expression after 48-h treatment. It also established the lentivirus-based expression of *Hes1*-shRNA II as a valuable strategy for genetic interference in the OC. As lentiviral vectors may provide lasting expression of the transgene compared to siRNA, they are suitable for future evaluation of efficacy and endurance of the effects of genetic interference *in vivo* on SC and HC phenotypes. Finally, it is important to highlight that the LIM operating in inner ear HC and SC involves other transcription factors, such as Hes5 and Hey1. These factors, in combination with *Hes1*, are believed to generate a graded regulation of HC production (15). Therefore, lentivirus-based approach tools to silence these factors in a combinatorial way are promising at improving the hearing function and quality of life of HL patients.

## Supplementary Material

Click here to view [pdf].

## References

[1] Mathers C, Smith A, Concha M (2000). Global burden of hearing loss in the year 2000. Global Burden of Disease.

[2] Atkinson PJ, Kim GS, Cheng AG (2019). Direct cellular reprogramming and inner ear regeneration. Expert Opin Biol Ther.

[3] Massucci-Bissoli M, Lezirovitz K, Oiticica J, Bento RF (2017). Evidence of progenitor cells in the adult human cochlea: sphere formation and identification of ABCG2. Clinics.

[4] Barboza LC, Lezirovitz K, Zanatta DB, Strauss BE, Mingroni-Netto RC, Oiticica J (2016). Transplantation and survival of mouse inner ear progenitor/stem cells in the organ of Corti after cochleostomy of hearing-impaired guinea pigs: preliminary results. Braz J Med Biol Res.

[5] Lanford PJ, Lan Y, Jiang R, Lindsell C, Weinmaster G, Gridley T (1999). Notch signaling pathway mediates hair cell development in mammalian cochlea. Nat Genet.

[B06] Zheng JL, Shou J, Guillemot F, Kageyama R, Gao WQ (2000). Hes1 is a negative regulator of inner ear hair cell differentiation. Development.

[7] Yamamoto N, Tanigaki K, Tsuji M, Yabe D, Ito J, Honjo T (2006). Inhibition of Notch/RBP-J signaling induces hair cell formation in neonate mouse cochleas. J Mol Med.

[8] Abdolazimi Y, Stojanova Z, Segil N (2006). Selection of cell fate in the organ of Corti involves the integration of Hes/Hey signaling at the Atoh1 promoter. Development.

[9] Du X, Li W, Gao X, West MB, Saltzman WM, Cheng CJ (2013). Regeneration of mammalian cochlear and vestibular hair cells through Hes1/Hes5 modulation with siRNA. Hear Res.

[10] Oiticica J, Barboza-Junior LC, Batissoco AC, Lezirovitz K, Mingroni-Netto RC, Haddad LA (2010). Retention of progenitor cell phenotype in otospheres from guinea pig and mouse cochlea. J Transl Med.

[11] Maass JC, Berndt FA, Cánovas J, Kukuljan M (2013). p27Kip1 knockdown induces proliferation in the organ of Corti in culture after efficient shRNA lentiviral transduction. J Assoc Res Otolaryngol.

[B12] Su YX, Hou CC, Yang WX (2015). Control of hair cell development by molecular pathways involving Atoh1, Hes1 and Hes5. Gene.

[13] Tomoko T, Itaru I, Ichiro T, Juichi I, Ryoichiro K (2011). Cooperative functions of hes/hey genes in auditory hair cell and supporting cell development. Dev Biol.

[B14] Wang B, Liu Y, Zhu X, Chi F, Zhang Y, Yang M (2011). Up-regulation of cochlear Hes1 expression in response to noise exposure. Acta Neurobiol Exp.

[15] Batts SA, Shoemaker CR, Raphael Y (2009). Notch signaling and Hes labeling in the normal and drug-damaged organ of Corti. Hear Res.

[16] Kempfle JS, Turban JL, Edge AS (2016). Sox2 in the differentiation of cochlear progenitor cells. Sci Reports.

[17] Oesterle EC, Chien WM, Campbell S, Nellimarla P, Fero ML (2011). p27(Kip1) is required to maintain proliferative quiescence in the adult cochlea and pituitary. Cell Cycle.

[18] Chen P, Segil N (1999). p27 (Kip1) links cell proliferation to morphogenesis in the developing organ of Corti. Development.

[19] Tateya T, Imayoshi I, Tateya I, Ito J, Kageyama R (2011). Cooperative functions of Hes/Hey genes in auditory hair cell and supporting cell development. Dev Biol.

[20] Du X, Cai Q, West MB, Youm I, Huang X, Li W (2018). Regeneration of cochlear hair cells and hearing recovery through hes1 modulation with siRNA nanoparticles in adult guinea pigs. Mol Ther.

